# AI-Assisted Detection for Early Screening of Acute Myeloid Leukemia Using Infrared Spectra and Clinical Biochemical Reports of Blood

**DOI:** 10.3390/bioengineering12040340

**Published:** 2025-03-26

**Authors:** Chuan Zhang, Jialun Li, Wenda Luo, Sailing He

**Affiliations:** 1 Center for Optical and Electromagnetic Research, National Engineering Research Center for Optical Instruments, College of Optical Science and Engineering, Zhejiang University, Hangzhou 310052, China; zhang.chuan@zju.edu.cn (C.Z.); 12430052@zju.edu.cn (J.L.); 2 Taizhou Hospital, Zhejiang University, Taizhou 318000, China; luowd@enzemed.com; 3 School of Information Science and Engineering, NingboTech University, Ningbo 315211, China; 4 Taizhou Agility Smart Technologies Co., Ltd., Taizhou 318000, China

**Keywords:** acute myeloid leukemia, early screening, blood analysis, infrared spectroscopy, artificial intelligence

## Abstract

Early detection and accurate diagnosis of leukemia pose significant challenges due to the disease’s complexity and the need for minimally invasive methods. Acute myeloid leukemia (AML) accounts for most cases of adult leukemia, and our goal is to screen out some AML from adults. In this work, we introduce an AI-enhanced system designed to facilitate early screening and diagnosis of AML among adults. Our approach combines the infrared absorption spectra of serum measured with attenuated total reflectance Fourier transform infrared spectroscopy (ATR-FTIR), which identifies distinctive molecular signatures in lyophilized serum, together with standard clinical blood biochemical test results. We developed a multi-modality spectral transformer network (MSTNetwork) to generate latent space feature vectors from these datasets. Subsequently, these vectors were assessed using a linear discriminant analysis (LDA) algorithm to estimate the likelihood of acute myeloid leukemia. By analyzing blood samples from leukemia patients and the negative control (including non-leukemia patients and healthy individuals), we achieved rapid and accurate prediction and identification of acute myeloid leukemia among adults. Compared to conventional methods relying solely on either FTIR spectra or biochemical indicators of blood, our multi-modality classification system demonstrated higher accuracy and sensitivity, ultimately achieving an accuracy of 98% and a sensitivity of 98%, improving the sensitivity by 12% (compared with using only biochemical indicators) or over 6% (compared with using only FTIR spectra). Our multi-modality classification system is also very robust as it gave much smaller standard deviations of the accuracy and sensitivity. Beyond improving early detection, this work also contributes to a more sustainable and intelligent healthcare sector.

## 1. Introduction

Leukemia is a malignant tumor of the blood system, characterized by the excessive production of abnormal white blood cells in the bone marrow or other hematopoietic tissues. This overproduction suppresses the formation of normal white blood cells, red blood cells, and platelets, leading to impaired hematopoiesis and the spread of leukemic cells to other tissues or organs [1,2]. Consequently, patients with leukemia frequently experience symptoms such as increased susceptibility to infections, easy bruising, fever, chills, and other flu-like symptoms [3,4]. Leukemia typically has an acute onset and is commonly diagnosed in children and young adults [5]. Statistics indicate that approximately 15,000 new cases of leukemia are reported annually in China, with the incidence rate among children increasing by about 2.8% each year [6], especially acute myeloid leukemia, which is a disease with extremely high mortality. Currently, the diagnosis of leukemia is a comprehensive and complex process that commonly involves hematological examinations, immunophenotype analysis, and genetic and molecular biological analyses of cells [7,8,9,10]. As a part of a routine blood examination, complete blood cell count has the advantages of easy operation, low cost, and can quickly provide information on the number of red blood cells, white blood cells, and platelets. However, its disadvantage lies in the lack of specificity, inability to directly identify leukemia cells, susceptibility to interference from various factors such as drugs and infections, and limited accuracy in leukemia screening, which may result in missed detection of some leukemia patients, i.e., false positive or false negative results. Flow cytometry has the advantages of multi-parameter analysis, high accuracy, and high sensitivity, and can quickly identify and classify leukemia cells and provide an important basis for leukemia diagnosis. However, its disadvantages lie in the complexity of operation, the need for professional equipment and operator support, and the high cost. It exhibits high accuracy in leukemia screening, can accurately identify leukemia cells, and has high specificity. PCR technology has the advantages of high sensitivity and specificity, and can amplify specific DNA or RNA sequences for the detection of leukemia-related genes. However, its drawbacks include cumbersome operation, strict control of experimental conditions, and the risk of contamination. It exhibits high accuracy in leukemia screening and can accurately amplify leukemia-related genes. Mass spectrometry has the advantages of high specificity and sensitivity, and can accurately identify and analyze specific compounds in samples, including leukemia-related metabolites. However, its disadvantages lie in the complexity of operation, the large amount of data generated that requires professional software for processing and analysis, the support of professional equipment and operators, and the high cost. It exhibits high accuracy in leukemia screening and can accurately identify leukemia-related metabolites. The sampling method of bone marrow smears is an invasive surgical procedure that imposes a significant burden on patients [11]. Minor complications may include pain, bleeding risks, potential wound infections, and nerve damage, while severe complications can lead to inadequate blood supply and bone marrow necrosis. Therefore, it is extremely important to choose detection methods that are simple and quick to perform and minimally invasive, carry low risks of complications, have high patient acceptability, and are widely applicable.

Spectroscopic detection technology is a rapid and highly sensitive quantitative method that plays a crucial role in identifying and characterizing material compositions. Among these techniques, infrared spectroscopy stands out due to its wide applicability, high specificity, non-destructive testing, rapid and efficient performance, and rich information content. FTIR has extensive applications and significant value in various fields, including scientific research, industrial production, and everyday life [12,13,14,15]. In clinical medicine, infrared spectroscopy is used to detect various cells or tissues by tracking the unique spectral characteristics of each molecule, such as proteins, nucleic acids, lipids, and other biomolecules [16,17,18]. Currently, infrared spectroscopy has been applied in the identification and diagnosis of various diseases, including Alzheimer’s disease, cervical cancer, gastric cancer, and leukemia [19,20]. Chaber et al. [21] employed FTIR spectroscopy for early screening of pediatric leukemia, with an identification accuracy of 85%. Zelig et al. [22] analyzed monocytes in peripheral blood using red-light spectroscopy, demonstrating that infrared spectroscopy holds great potential for pre-screening and follow-up in pediatric leukemia. However, the diagnostic accuracy of these methods remains relatively low, making the development of more accurate disease identification methods urgently needed.

Artificial intelligence, as an emerging technology [23], is gradually transforming the way cancer detection is conducted due to its capability to extract high-level abstract information. For example, Du et al. [24] constructed a one-dimensional convolutional neural network (1D-CNN) aimed at processing one-dimensional Raman spectroscopy data, achieving a 94.5% accuracy in identifying and classifying four types of cancer, including gastric, colon, rectal, and lung cancer. A handheld Raman spectrometer was designed in [25], and integration with a convolutional neural network allowed precise classification of pancreatic cancer, with sensitivity, specificity, and accuracy all exceeding 95%. While previous studies achieved good results, they typically focused on only one spectral modality. In some other types of diseases, multi-modality models have shown stronger performance compared to unimodal models. In [26], the fusion of infrared spectroscopy, Raman spectroscopy, and the first derivative of Raman spectroscopy increased precision by nearly 20% compared to unimodal approaches, demonstrating the significant potential of multi-modality models in medical diagnosis. In [27], the complementary analysis of FTIR and Raman spectra of serum for classifying healthy controls, gliomas, non-small cell lung cancer, and esophageal cancer showed that the accuracy of both low-level fusion models and feature fusion models improved by about 10% compared to single spectral models.

Currently, although clinical serum biochemical testing technologies have drawbacks such as complex operation, long detection times, and low sensitivity, they remain irreplaceable. While Raman spectroscopy and FTIR spectroscopy can reflect the content of various compounds at the molecular level, their signals often overlap. By accurately measuring the biochemical components in blood, serum biochemical testing provides valuable diagnostic information and treatment guidance to clinicians, aiding in the early detection and treatment of diseases, thereby improving patient outcomes and quality of life. Therefore, it is particularly important to develop an auxiliary method that can enhance the sensitivity of serum testing.

In this study, we developed an artificial intelligence-based system and method for early screening and diagnosis of acute myeloid leukemia among adults, utilizing infrared absorption spectroscopy and standard clinical biochemical blood reports. Specifically, we innovatively propose a multi-modality transformer architecture named MSTNetwork, which achieves adaptive fusion of modal features through an improved attention mechanism. Compared to existing transformer network structures [28], our model, by designing a dual-path attention layer, can dynamically generate attention weight matrices for both intra-modal and inter-modal interactions, enabling hierarchical feature interaction. This end-to-end learning paradigm eliminates the need for manually designed feature fusion rules and significantly enhances the model’s compatibility with heterogeneous modal data. By analyzing and comparing blood samples from acute myeloid leukemia patients, non-leukemia patients, and healthy individuals, we achieved rapid and accurate prediction and identification of acute myeloid leukemia. When using only the biochemical blood test reports or only the FTIR data for serum sample analysis, the prediction accuracies were 93% (with a sensitivity of 0.86 ± 0.09 due to the large false negative rate) and 93% (with the sensitivity of 0.92 ± 0.03), respectively. However, the intelligent early screening system developed using both the biochemical blood test reports and FTIR data improved the prediction accuracy for acute myeloid leukemia serum samples to 98% (with the sensitivity of 0.98 ± 0.02), which is crucial for the rapid screening and auxiliary diagnosis of acute myeloid leukemia. Our multi-modality approach not only improves the sensitivity (related to the false negative rate) by 12% (compared with using only biochemical indicators) or over 6% (compared with using only FTIR spectra) but also gives much smaller standard deviations of the accuracy and sensitivity, indicating that our multi-modality approach is accurate and robust.

## 2. Materials and Methods

### 2.1. Materials

**Figure 1:**
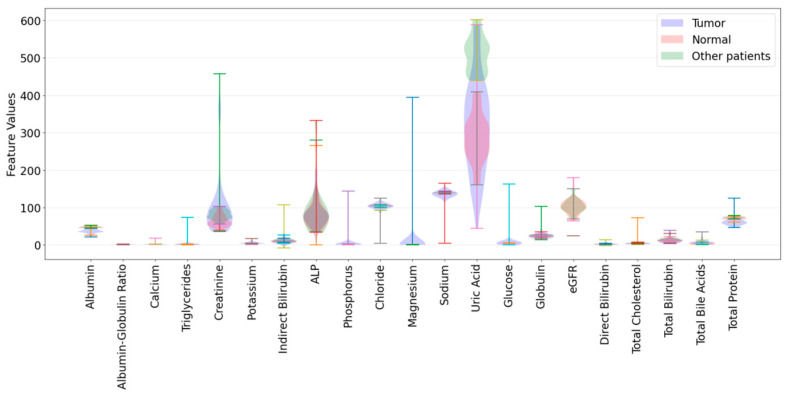
Feature value distribution of biochemical indicator data for tumor, normal, and other patients.

This study was approved by the Ethics Committee of Zhejiang Taizhou Enze Medical Center (Group). A total of 70 patients and 24 healthy individuals participated in the research. The patient group included 36 acute myeloid leukemia patients and 24 individuals with other non-leukemia diseases, comprising 10 with hyperuricemia, 7 with gout, and 7 with rheumatoid arthritis. Leukemia diagnoses (as the ground truth) were confirmed through clinical methods such as blood, bone marrow, cellular, or morphological analyses. Blood samples were collected from each patient at different time points. Patients with other diseases were also diagnosed through clinical methods. Blood from healthy individuals was provided by the physical examination center. Blood was collected in anticoagulant tubes and centrifuged at 3000 rpm/min within two hours to obtain serum, which was then stored at −80 °C. All participants’ biochemical blood test reports were provided by Zhejiang Taizhou Enze Medical Center (Group). Images of positive and negative biochemical indicator data are shown in Figure 1. The graph indicates that the levels of compounds such as albumin, total protein, and lactate dehydrogenase differed significantly between positive and negative cases, playing an important role in our AI classification. Compounds like potassium and uric acid show smaller variations, making their contribution to AI classification relatively minimal.

### 2.2. Methods

#### 2.2.1. Sample Processing

All blood was collected in anticoagulant tubes and centrifuged at 3000 rpm/min within two hours to obtain serum, which was then stored at −80 °C for subsequent use. Throughout the experiment, the stored serum samples were subjected to freeze-drying. Specifically, 100 µL of each serum sample was freeze-dried for 48 h using an LGJ-12A freeze dryer (Beijing Sihuan Qihang Technology Co., Ltd., Beijing, China) and subjected to FTIR detection.

#### 2.2.2. FTIR Spectroscopic Measurement

**Figure 2:**
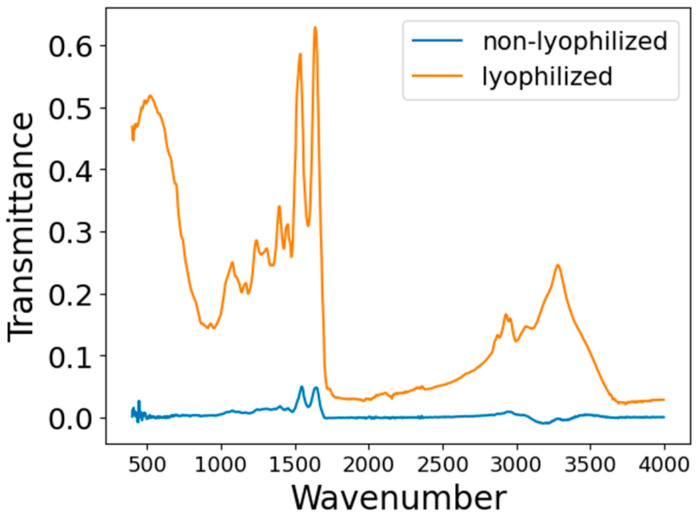
Comparison of infrared spectra of lyophilized and non-lyophilized samples.

**Figure 3:**
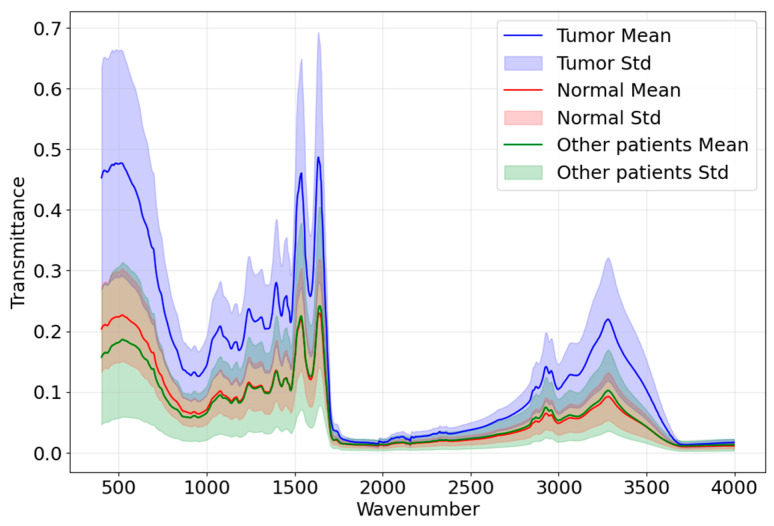
FTIR curves for positive and negative cases. The blue solid line and red solid line represent the mean values of infrared spectrum curves for positive and negative samples, respectively; the blue range and the red range show the standard deviations of the infrared spectrums for positive and negative samples, respectively.

In this experiment, preserved serum samples were subjected to freeze-drying. The resulting serum solids were then analyzed by FTIR to eliminate water interference in the spectra. The infrared spectra of lyophilized and non-lyophilized samples are shown in Figure 2. Infrared spectral data were obtained using an INVENIO S FTIR spectrometer (Bruker, Beijing Scientific Technology Co., Ltd., Beijing, China) equipped with a diamond attenuated total reflectance (ATR) accessory. All serum spectra were recorded within the infrared wavenumber range of 400–4000 cm^−^¹ with a spectral resolution of 2 cm^−^¹. Prior to each measurement, the detection area was cleaned with anhydrous ethanol, and air was used as the background. Subsequently, clean tweezers were used to take an appropriate amount of freeze-dried serum sample and place it on the diamond crystal attachment, making it adhere tightly to the attachment crystal. Then, using the infrared spectrometer, we collected the infrared spectrum of each sample. Each serum sample was sampled and tested at least five different locations, and the average value used. To avoid sample or environmental contamination, all consumables and operations were consistent. The FTIR spectral curves for positive and negative samples are shown in Figure 3.

## 3. Algorithm

### 3.1. MSTNetwork

Linear discriminant analysis (LDA), as a classic machine learning algorithm, performs well in certain tasks, especially when data are linearly separable and the feature dimensionality is moderate. However, LDA often relies excessively on the linear separability of the feature space, which is particularly pronounced when dealing with high-dimensional data. In high-dimensional settings, the number of samples is typically much smaller than the number of features, causing singular matrices when computing the within-class scatter matrix in LDA, thereby affecting the model’s stability and discriminative ability. Additionally, when handling multi-modality datasets, LDA lacks a natural mechanism to integrate heterogeneous information, typically requiring separate processing and feature extraction for each modality before integration. This process is not only complex and susceptible to information loss but also may increase integration difficulties due to heterogeneity between modalities, limiting the enhancement of overall classification performance.

**Figure 4:**
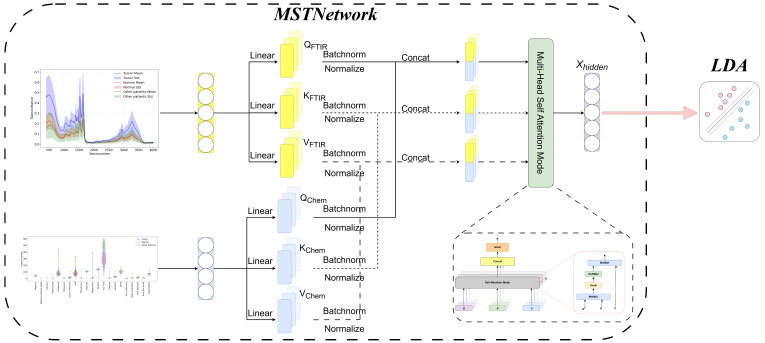
The structure of multi-modality spectral transformer network (MSTNetwork).

To address the aforementioned issues, we propose a novel transformer-based neural network, the multi-modality spectral transformer network (MSTNetwork), to encode the raw data before inputting them into the LDA model for sample classification, as illustrated in Figure 4. Generally, medical data are characterized by high dimensionality, high noise levels, and complex dependencies. Therefore, applying a transformer network during data processing can effectively capture global dependencies and intricate features. The transformer network utilizes a self-attention mechanism to establish long-range dependencies across different dimensions, identifying important features related to diseases or specific physiological processes. Unlike traditional convolutional neural networks, transformers do not rely on local perception but instead employ a multi-head attention mechanism to simultaneously focus on multiple subspaces of the data, thereby gaining a more comprehensive understanding of the data’s inherent structure and preventing information loss. Additionally, the parallel computing capabilities of transformers enhance the model’s training efficiency, making it suitable for processing large-scale medical data.

Specifically, the input data of MSTNetwork consists of two types: infrared spectral data ($X_{FTIR}\in R^{d_{FTIR}}$) and biochemical data ($X_{Chem}\in R^{d_{Chem}}$), where $d_{FTIR}$ and $d_{Chem}$ represent the lengths of the infrared spectral data and biochemical data, respectively. Since infrared spectral data and biochemical data inherently possess different physical properties, such as absorption rates at various wavelengths and the concentrations of various biochemical indicators, the data are not passed through a LayerNorm layer. Instead, the two types of data are directly encoded into *Q*, *K*, and *V* by fully connected layers, separately, resulting in $Q_{FTIR},\;K_{FTIR},{\;V}_{FTIR}\in R^{d_{1}},\;Q_{Chem},\;K_{Chem},{\;V}_{Chem}\in R^{d_{2}}$, where $d_{1},d_{2}$ represent the lengths after encoding the infrared spectral data and biochemical data and are set as 32 and 8 in this paper, respectively. A batch normalization operation is performed on the different data of each modality to stabilize the input distribution of subsequent modules, thereby reducing the model’s dependence on initialization characteristics and enabling better convergence. Its specific expression is as follows:

$$\mu_{B}=\frac{1}{m}\sum_{i=1}^{m}x_{i}$$

$$\sigma_{B}^{2}=\frac{1}{m}\sum_{i=1}^{m}{\left(x_{i}-\mu_{B}\right)}^{2}$$

$$\hat{x_{i}}=\frac{x_{i}-\mu_{B}}{\sqrt{\sigma_{B}^{2}+\varepsilon }}$$

$$y_{i}=\gamma \hat{x_{i}}+\beta$$

where $x_{i}$ represents the one-dimensional infrared spectral data or biochemical data, and *m* represents the batch size.

Infrared spectral and biochemical data are two highly correlated modalities that reveal cancer-related pathological features from the perspectives of molecular compound characterization and biological metabolic processes, respectively. In single-modality analysis scenarios, data from the other modality can provide complementary lesion clues; for example, infrared spectra can locate abnormal molecular groups, while biochemical indicators can reflect abnormalities in metabolic pathways. To fully utilize the potential synergistic correlations between modalities, $Q_{FTIR},{\;K}_{FTIR},{\;V}_{FTIR}$ are connected with $Q_{Chem},{\;K}_{Chem},{\;V}_{Chem}$, respectively, and feature extraction for individual modalities and the correlations between multiple modalities are performed through a multi-head attention mechanism:

$$Attention(Q,K,V)=softmax(\frac{Q^{T}K}{\sqrt{d_{k}}})V$$

where Q represents the connection of $Q_{FTIR}$ and $Q_{Chem}$; K represents the connection of $K_{FTIR}$ and $K_{Chem}$; and V represents the connection of $V_{FTIR}$ and $V_{Chem}$.

Finally, the data are normalized as the final output of MSTNetwork.

### 3.2. Contrastive Learning

Contrastive learning is a deep learning method that learns feature representations by comparing the similarities and differences between various samples. The core idea is to pull similar samples (positive samples) closer together and push different samples (negative samples) apart, enabling the model to develop feature representations that are sensitive to the differences between samples. First, a set of infrared spectral data and biochemical data is input into the MSTNetwork, which reduces their dimensionality from $R^{d_{FTIR}+d_{Chem}}$ to $R^{d_{1}+d_{2}}$ to obtain a two-dimensional matrix. Then, the Euclidean distances between different row vectors in X are calculated to obtain a distance matrix $M\in R^{batchsize*batchsize}$, which is inherently symmetric and satisfies $M_{i,j}=\sqrt{\sum_{l=1}^{d_{1}+d_{2}}{\left(X_{i,l}-X_{j,l}\right)}^{2}}$. Then, we define the following loss function according to the ground truth:

$$L_{contrastive}(x_{i},x_{j},\theta )=l[y_{i}=y_{j}]{\left||\theta (x_{i})-\theta (x_{j})|\right|}_{2}+l[y_{i}\neq y_{j}]{\cdot max(0,\epsilon -||\theta (x_{i})-\theta (x_{j})||}_{2})$$

where $x_{i}$ represents the i-th sample; $y_{i}$ represents the i-th ground truth; $l[y_{i}=y_{j}]$ equals 1 when the i-th sample and j-th sample belong to the same category and 0 otherwise; θ represents the weight parameters of MSTNetwork; and ϵ is a hyperparameter which equals 0.5. The core idea behind the setup of the aforementioned process is to use a feature extraction network to reduce the dimensionality of the raw data and learn useful features. Then, through a maximum margin loss function, the distance between samples of the same class is minimized, while the distance between samples of different classes is maximized. The choice of batch size affects the final training outcome. A smaller batch size can make it difficult for the model to comprehensively distinguish between positive and negative samples, hindering convergence, whereas a larger batch size may lead to overfitting. In this study, the batch size is set to 300. During training, the Adam optimizer is used with a learning rate of 10^−3^. Additionally, PyTorch’s StepLR scheduler is employed with a patience of 60 and a gamma of 0.5. This means that every 60 epochs, the learning rate is reduced by 50%, helping the model escape local minima and accelerate convergence. The total number of training epochs is set to 240.

## 4. Results

### 4.1. The Visualization of Contrastive Learning

**Figure 5:**
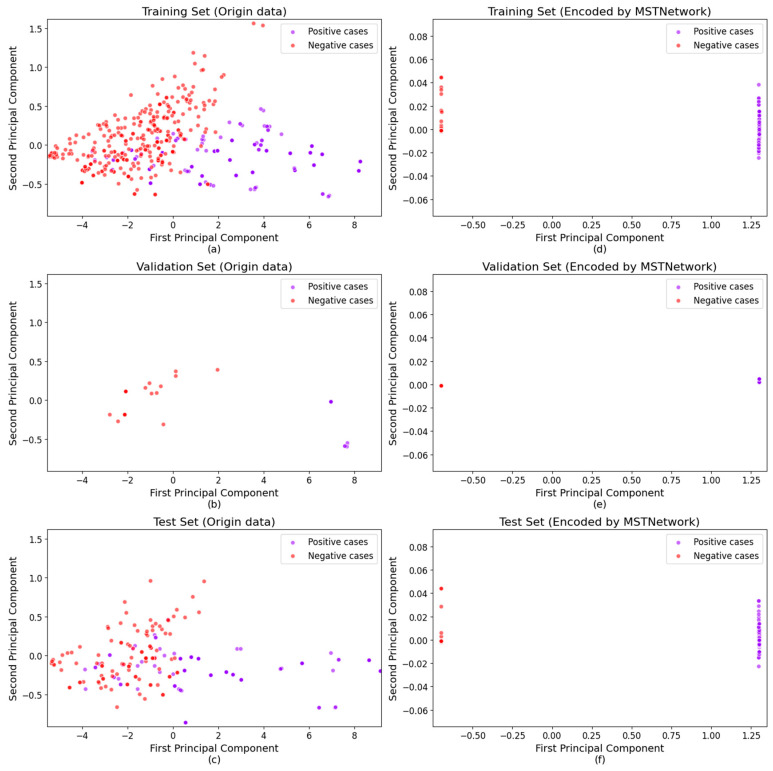
(**a**) Distribution of the training set after MSTNetwork encoding; (**b**) Distribution of the validation set after MSTNetwork encoding; (**c**) Distribution of the test set after MSTNetwork encoding; (**d**) Distribution of the original training data; (**e**) Distribution of the original validation data; (**f**) Distribution of the original test data.

To visually demonstrate the feature extraction capability of the pre-trained model, the PCA algorithm is applied to both the data processed by the feature extraction network and the raw data for dimensionality reduction. The prediction results are shown accordingly in Figure 5, from which it is evident that the original data have a high degree of overlap between different classes. Therefore, using an end-to-end deep learning model for prediction may require a very deep network to capture the features, resulting in poor model robustness. In contrast, after pre-training with a contrastive learning approach, different types of data are distinctly separated, significantly reducing the classification difficulty for subsequent predictive models. The distances between different classes are relatively large, which enhances the model’s robustness.

### 4.2. The Selection of the Hyperparameter

**Figure 6:**
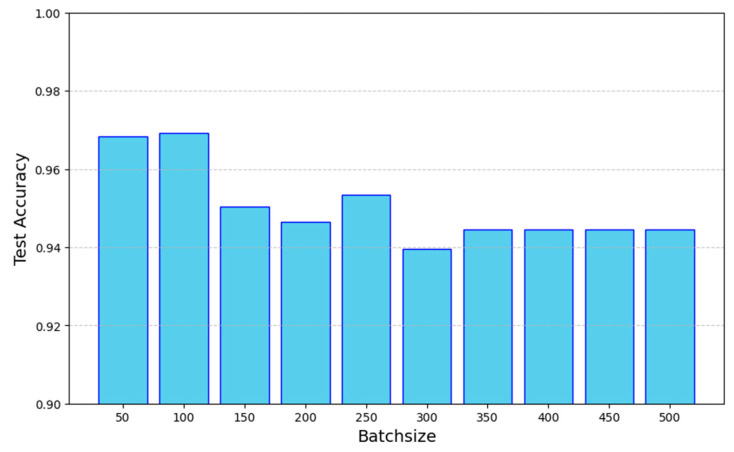
The accuracy corresponding to different batch sizes.

As illustrated in Section 3.2, the performance of the MSTNetwork model is influenced by the batch size. As the batch size increases in the training period, the model will differentiate or merge features from more pairs of data, thereby increasing its fit to the training set. A batch size that is too large can cause the model to learn more about the feature differences or relationships among the samples, while a smaller batch size may hinder the model’s ability to effectively extract features. To determine an appropriate batch size, we conducted experiments with increments of 50. The results, as shown in Figure 6, met our expectations, leading us to select a batch size of 100, which yielded the highest accuracy, for subsequent experiments.

### 4.3. Ablation Study

In this section, we demonstrate how multi-modality data enhance overall classification accuracy. To mitigate interference from model initialization and test set variations, we conducted 10 parallel experiments. For each experiment, the dataset was divided into training, validation, and test sets at a 7:0.5:2.5 ratio, with five random model initializations performed. The final accuracy was evaluated as the average of 50 total trials. In our experiments, we explored different data split ratios. Our findings suggest that a 5% validation set provided reliable feedback during the training and tuning phases without significantly impacting the model’s generalization performance. Meanwhile, the larger test set (25%) was critical for ensuring a robust and unbiased evaluation of final model performance. In practical applications, a single patient may contribute multiple serum samples. Consequently, to ensure the integrity of our data analysis, it is important to prevent any overlap where samples from the same individual appear in both the training and testing datasets. By splitting our dataset based on individual subjects rather than on a per-sample basis, we mitigate the risk of data contamination and bolster the reliability of our model evaluation. This approach not only preserves the independence of the training and testing sets, but it also contributes to a more accurate assessment of the model’s generalizability in clinical settings. When assessing the accuracy of a method, the quantities used include the accuracy, sensitivity, and specificity, which are defined as in the literature [29].

$$Accuracy=\frac{TP+TN}{TP+TN+FP+FN}\;Sensitivity=\frac{TP}{TP+FN}\;Specificity=\frac{TN}{TN+FP}$$

where TP,TN, FP,andFN denote the rates of “true positive”, “true negative”, “false positive” and “false negative”, respectively. When the numbers of positive and negative samples are approximately equal (i.e., TP + FN TN + FP), which was the case in our test dataset, the following relationship holds:

Accuracy≈(Sensitivity+Specificity)/2

Thus, in this work we used the accuracy and sensitivity to access the accuracy of our trained AI algorithm on the test dataset.

**Figure 7:**
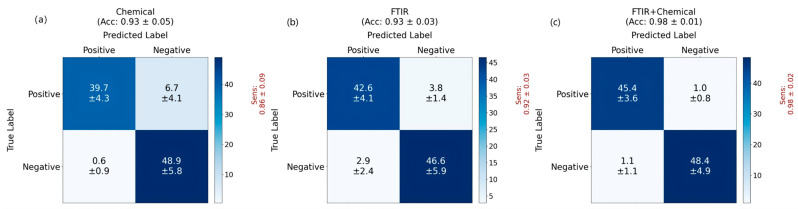
The results of the datasets containing acute myeloid leukemia patients and the control group (including healthy individuals and other patients): (**a**) Confusion matrix using only biochemical data; (**b**) Confusion matrix using only the infrared spectroscopy data; (**c**) Confusion matrix using both infrared spectroscopy and biochemical data.

**Figure 8:**
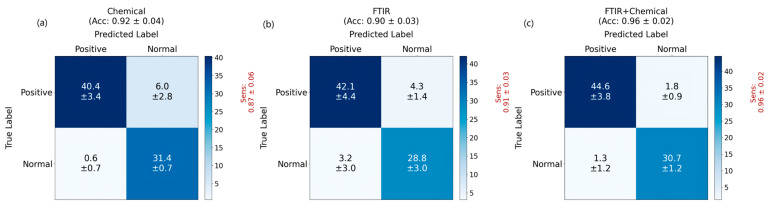
The results of the datasets containing acute myeloid leukemia patients and the control group (only healthy individuals): (**a**) Confusion matrix using only biochemical data; (**b**) Confusion matrix using only the infrared spectroscopy data; (**c**) Confusion matrix using both infrared spectroscopy and biochemical data.

As shown in Figure 7, the application of combined infrared spectroscopy and biochemical indicators has demonstrated impressive performance in distinguishing acute myeloid leukemia patients from the negative control (including some non-leukemia patients and healthy individuals). However, using only biochemical indicators (with 36 standard major biochemical indicators) exhibited a poor classification performance of 93% accuracy and 86% sensitivity (due to a large false negative rate). Using only infrared spectroscopy achieved an accuracy of 93%, along with a sensitivity of 92% (and a specificity of 94%), leveraging the vibrational energy information from diverse molecules. When both infrared spectroscopy and biochemical indicators were effectively integrated, MSTNetwork further enhanced diagnostic performance, elevating accuracy to 98% and sensitivity to 98%. Our multi-modality approach not only improved the sensitivity (related to the false negative rate) by over 12% (compared with using only biochemical indicators) or over 6% (compared with using only infrared spectroscopy), but also gave a much smaller standard deviation of the sensitivity (indicating that our approach is very robust). These findings underscore the efficacy of combining data from different measurement methods, offering a promising solution that refines diagnostic accuracy and efficiency in clinical settings. Figure 8 further illustrates the classification of acute myeloid leukemia patients and healthy individuals, showing that the accuracy and sensitivity metrics all remained at a high level.

## 5. Conclusions

In this study, we developed an AI-based early screening diagnostic system and method for acute myeloid leukemia, utilizing an infrared absorption spectroscopy detection system combined with standard biochemical blood test reports. FTIR analysis can provide the molecular structure and chemical bonding information of the sample, while biochemical analysis can reveal the function and activity of biomolecules. The combination of these two analytical methods enables more comprehensive information related to the diseases. By performing infrared detection on lyophilized serum and integrating results from routine biochemical indicators, we applied an AI algorithm to analyze the data, achieving an acute myeloid leukemia screening accuracy of 98% and a sensitivity of 98%. Compared to using only FTIR analysis (accuracy of 93%, sensitivity of 92%) or biochemical analysis alone (accuracy of 93%, sensitivity of 86%), this system demonstrates an enhanced classification accuracy, enabling rapid and precise acute myeloid leukemia prediction. Furthermore, current leukemia diagnoses largely depend on bone marrow biopsies, which are highly invasive and cause significant discomfort for patients. Routine blood tests have limited sensitivity and specificity, leading to potential misdiagnoses or missed diagnoses, while also being time-consuming and costly. The infrared absorption spectroscopy system requires only a few hundred microliters of serum per test, which helps alleviate both the psychological and physical stress on patients. This study fully leveraged the advantages of infrared absorption spectroscopy in providing “molecular-level fingerprint” information and harnessed the robust capabilities of AI algorithms for analyzing complex data. It presents a novel solution for AI-assisted early screening and clinical diagnosis of leukemia. Although the method constructed in this study has high accuracy and reliability compared to other leukemia screening methods, and is relatively low-cost, there are still some areas that need improvement. Firstly, the use of freeze-drying treatment for samples takes a longer time from collection to detection of serum samples, which may to some extent affect the efficiency of screening. To shorten this time, we will explore more efficient and rapid sample processing techniques to accelerate the screening process. Secondly, this study only conducted a simple binary classification, distinguishing acute myeloid leukemia from the control adult group (healthy and patients of non-leukemia diseases). AML accounts for 60%~70% of adult acute leukemia. Our goal was to screen out some AML from adults. Thus, the leukemia patients in our test dataset were all adults, aged between 40–70 years old, and belonged to AML. In order to have a more comprehensive understanding of the disease status, future research can further refine the classification to include all leukemia subtypes for all ages. This will help us more accurately identify disease characteristics, develop more personalized treatment plans, and provide stronger support for clinical diagnosis and treatment.

## Data Availability

The data presented in this study may be available from the corresponding author upon special request.
